# Multi-Targeting Anticancer Activity of a New 4-Thiazolidinone Derivative with Anti-HER2 Antibodies in Human AGS Gastric Cancer Cells

**DOI:** 10.3390/ijms24076791

**Published:** 2023-04-05

**Authors:** Agnieszka Gornowicz, Roman Lesyk, Robert Czarnomysy, Serhii Holota, Yulia Shepeta, Bożena Popławska, Magdalena Podolak, Wojciech Szymanowski, Krzysztof Bielawski, Anna Bielawska

**Affiliations:** 1 Department of Biotechnology, Faculty of Pharmacy, Medical University of Bialystok, 15-089 Bialystok, Polandmagdalena.podolak@sd.umb.edu.pl (M.P.); wojtekszymanowski@wp.pl (W.S.); anna.bielawska@umb.edu.pl (A.B.); 2 Department of Biotechnology and Cell Biology, Medical College, University of Information Technology and Management in Rzeszow, Sucharskiego 2, 35-225 Rzeszow, Poland; dr_r_lesyk@org.lviv.net; 3 Department of Pharmaceutical, Organic and Bioorganic Chemistry, Danylo Halytsky Lviv National Medical University, Pekarska 69, 79010 Lviv, Ukraine; golota_serg@yahoo.com; 4 Department of Synthesis and Technology of Drugs, Faculty of Pharmacy, Medical University of Bialystok, 15-089 Bialystok, Poland; robert.czarnomysy@umb.edu.pl (R.C.); kbiel@umb.edu.pl (K.B.); 5 Department of Pharmaceutical Chemistry, National Pirogov Memorial Medical University, Pirogov 56, 21018 Vinnytsya, Ukraine; shepeta.yulia@gmail.com

**Keywords:** synthesis, 4-thiazolidinones, pertuzumab, trastuzumab, gastric cancer, apoptosis, autophagy, combination therapy

## Abstract

Combining chemotherapy with immunotherapy still remains a regimen in anticancer therapy. Novel 4-thiazolidinone-bearing hybrid molecules possess well-documented anticancer activity, and together with anti-HER2 antibodies, may represent a promising strategy in treating patients with gastric cancer with confirmed human epidermal growth factor receptor 2 (HER2) expression. The aim of the study was to synthesize a new 4-thiazolidinone derivative (Les-4367) and investigate its molecular mechanism of action in combination with trastuzumab or pertuzumab in human AGS gastric cancer cells. AGS cell viability and antiproliferative potential were examined. The effect of the tested combinations as well as monotherapy on apoptosis and autophagy was also determined. Metalloproteinase-2 (MMP-2), intercellular adhesion molecule 1 (ICAM-1), pro-inflammatory and anti-inflammatory cytokine concentrations were also demonstrated by the ELISA technique. We proved that pertuzumab and trastuzumab were very effective in increasing the sensitivity of AGS gastric cancer cells to novel Les-4367. The molecular mechanism of action of the tested combination is connected with the induction of apoptosis. Additionally, the anticancer activity is not associated with the autophagy process. Decreased concentrations of pro-inflammatory cytokines, MMP-2 and ICAM-1—were observed. The novel combination of drugs based on anti-HER2 antibodies with Les-4367 is a promising strategy against AGS gastric cancer cells.

## 1. Introduction

Gastric cancer (GC) represents a major public health problem and ranks as the second leading cause of cancer-related deaths worldwide. A number of etiologic factors predispose to gastric cancer development. *Helicobacter pylori* and Epstein-Barr virus are very important risk factors. Among environmental factors, we can emphasize the high intake of salted, pickled, or smoked food, low intake of fruits and vegetables, and tobacco smoking [1]. Individuals with a body mass index (BMI) of 30 or higher have an increased risk of esophagogastric junction cancers [2]. A positive family history is also a predisposing factor for gastric cancer [3]. Other potential risk factors include gastroesophageal reflux disease, radiation, poor oral hygiene, tooth loss, and eating pickled vegetables [4,5,6,7,8].

Ceasing smoking, reducing salt intake, increasing fruit and vegetable intake, and physical activity represent strategies for preventing the incidence of GC [9,10].

The choice of treatment depends on many factors such as tumor size and location, disease stage, and the patient’s general health. Treatment includes surgery, chemotherapy, and radiotherapy. Targeted therapy is another choice for gastric cancer treatment [1]. Researchers are focused on looking for novel HER2-targeted agents (ERBB2). HER2 amplification/overexpression is found not only in breast and gastric cancer but is also present in colon, biliary, and bladder cancers [11]. It drives tumorigenesis through the activation of oncogenic downstream signaling, such as phosphatidylinositol-3 kinase/ protein kinase B/ mechanistic target of rapamycin (PI3K/Akt/mTOR) and mitogen-activated protein kinase (MAPK) [12].

Most patients with advanced gastric cancer receive doublet combination therapy with platinum and fluoropyrimidine. Trastuzumab was approved by the Food and Drug Administration (FDA) for HER2-positive metastatic gastric/gastroesophageal junction cancers in combination with fluoropyrimidine (5-fluorouracil or capecitabine) and cisplatin. The TOGA trial proved that the median overall survival of patients who received trastuzumab with fluoropyrimidine and cisplatin improved compared with patients treated with chemotherapy alone [13].

The next choice in later therapy includes docetaxel, paclitaxel, or irinotecan monotherapy, trifluridine and tipiracil, and fluorouracil plus irinotecan. Ramucirumab is used in combination with paclitaxel as second-line therapy [14]. Nivolumab or pembrolizumab are anti-PD-1 third-line treatments. The efficacy of anti-PD-1 monoclonal antibodies was analyzed in the ATTRACTION-2 and KEYNOTE-059 trials [15,16].

4-thiazolidinone-bearing hybrid molecules possess anticancer activity via various mechanisms of action, such as apoptosis induction, cell cycle arrest, and reactive oxygen species (ROS) induction. This group of compounds includes representants that act as kinase, tubulin, and carbonic anhydrase inhibitors [17]. Recently, we presented the multi-targeting mode of action of 2-{5-[(Z,2Z)-2-chloro-3-(4-nitrophenyl)-2-propenylidene]-4-oxo-2-thioxothiazolidin-3-yl}-3-methylbutanoic acid (Les-3331). We demonstrated that a novel 4-thiazolidinone derivative possesses high cytotoxic and antiproliferative activity in MCF-7 and MDA-MB-231 breast cancer cells. Its molecular mechanism of action is associated with apoptosis induction, where mitochondrial membrane potential decreased, and increased caspase-9 and caspase-8 concentrations were observed. Les-3331 decreased autophagic marker (LC3A, LC3B, and Beclin-1) concentrations in the tested cell lines as well as reduced topoisomerase II concentrations [18].

Unfortunately, the prognosis for patients with advanced GC is poor, with limited survival, so novel beneficial therapeutic strategies have to be developed to improve life expectancy for patients with HER2-positive disease. The aim of the study was to examine the effectiveness of the combination of trastuzumab or pertuzumab with a new pyrazoline-bearing 4-thiazolidinone derivative (Les-4367) against AGS gastric cancer cells compared with monotherapy based on the aforementioned heterocyclic derivative. Pyrazoline-bearing hybrids are attractive objects for the design of potential anticancer agents among 4-thiazolidinone-based hybrid molecules [19]. So, early on, the huge anticancer potential of 5-ene-4-thiazolidinone derivatives with a pyrazoline core linked by an enamine linker was identified and reported [20]. Derivatives with such a chemotype were found to be highly active anticancer agents with mitochondria-dependent apoptosis as the main mode of action, as well as inducing cell arrest, cell division inhibition, and ROS production activation.

Les-4367 is a representative of the pyrazoline-thiazolidinones mentioned above with established anticancer potency, which we described in our previous paper [20]. At first, we checked the effect of Les-4367 alone and in combination with anti-HER2 monoclonal antibodies on viability and DNA biosynthesis. Then, we analyzed the effect of the tested strategies on apoptosis and autophagy induction. Additionally, MMP-2, ICAM-1 as well as interleukin-6 (IL-6), interleukin-8 (IL-8), and interleukin-10 (IL-10) concentrations were measured. 

## 2. Results

### 2.1. Synthesis of 4-Thiazolidinone-Based Derivative Les-4367

**Scheme 1:**
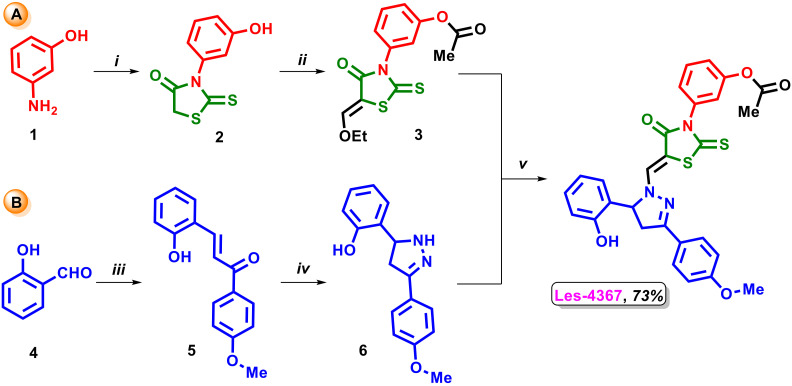
Synthesis of target 4-thiazolidinone-based derivative **Les-4367** (A. synthesis of 3-(5-ethoxymethylene-4-oxo-2-thioxothiazolidin-3-yl)phenyl acetate **3**; B. synthesis of 5-(2-hydroxyphenyl)-3-(4-methoxyphenyl)-4,5-dihydro-1H-pyrazole **6**). Reagents and conditions: (i) **1** (10 mmole), CS(SCH_2_COOH)_2_ (10 mmole), C_2_H_5_OH:H_2_O, reflux, 5 h; (ii) **2** (10 mmole), HC(OC_2_H_5_)_3_ (10 mmole), Ac_2_O, reflux, 3 h; (iii) **4** (10 mmole), 4-methoxyacetophenone (10 mmole), NaOH (10 mmole); (iv) **5** (10 mmole), NH_2_-NH_2_×H_2_O (10 mmole), KOH (10 mmole), C_2_H_5_OH; (v) **3** (10 mmole), **6** (10 mmole), C_2_H_5_OH, reflux, 2 h.

The synthetic design for Les-4367 included two key routes with the application of a scaffold-hybridization approach at the final step, as presented in Scheme 1. Initially, 4-thiazolidinone derivative **2** was easily obtained using Holmberg’s protocol (i) from m-aminophenol **1**. The treatment of derivative **2** by triethyl orthoformate in acetic anhydride medium (ii) led to obtaining derivative **3**. Diarylpyrazoline **6** was synthesized in two steps, starting from salicylic aldehyde in reaction with 4-methoxyacetophenone (iii), followed by the heterocyclization of the obtained product **5** by the action of hydrazine hydrate (iv).

The target 4-thiazolidinone derivative Les-4367 was synthesized with satisfactory yields and purity in the interaction of compounds **3** and **6** under reflux in an ethanol medium for 30 min. The structure of the novel synthesized derivative **Les-4367** was confirmed by ^1^H, ^13^C NMR, and LC-MS spectrometry (Figures S1 and S2, Supplementary Materials). In the ^1^H NMR spectra, the proton signal at the C-5 double bond appeared as a singlet at 7.48 ppm. The pyrazoline fragment of compound **Les-4367** showed characteristic patterns of the AMX system for CH_2_-CH protons with three doublets of doublets at 3.51, 3.86, and 5.69 ppm, and the appropriate spin-spin coupling constants.

### 2.2. Viability

Preliminary cytotoxic activity studies for Les-4367 were performed on six cancer cell lines—SK-BR-3, HCC1954, MDA-MB-231, HT-29, DLD-1, and AGS. The lowest IC50 was detected in human AGS gastric cancer cells, which also have confirmed HER2 expression (Table 1). The preliminary study confirmed that Les-4367 showed the highest cytotoxic activity in gastric cancer cells; therefore, it was subjected to a broader analysis for its molecular mechanism of action in monotherapy and in combination with anti-HER2 antibodies (pertuzumab, trastuzumab). 

**Figure 1:**
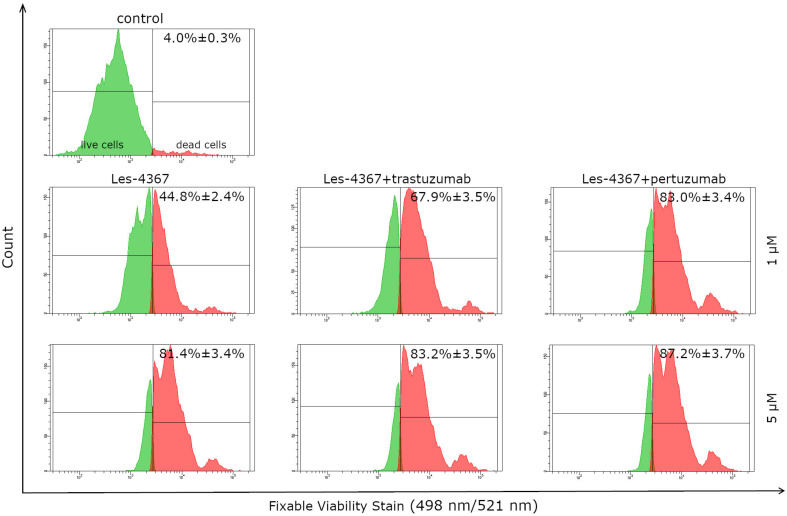
The effect of Les-4367 alone and its combination with trastuzumab or pertuzumab (Les-4367 + trastuzumab, Les-4367 + pertuzumab) on the viability of AGS human gastric cancer cells after 24 h incubation. The numerical value in the upper part of the histogram = the percentage of dead cells. Green part of histogram- live cells, red part of histogram- dead cells. The obtained results are presented as mean values ± SD obtained from three independent experiments (n = 3) performed in duplicate.

The cytotoxic activity of the synthesized 4-thiazolidinone derivative Les-4367 alone and in combination with trastuzumab and pertuzumab was investigated against human AGS gastric cancer cells. Figure 1 shows that AGS survival was significantly inhibited after 24 h of incubation with both the compound Les-4367 alone and in combination with monoclonal antibodies in a concentration-dependent manner. The results showed that Les-4367 alone at concentrations of 1 μM and 5 μM caused 44.8% ± 0.3% and 81.4% ± 3.4% of cells to die, respectively. In the case of combination therapy, we observed: Les-4367 with trastuzumab, 67.9% ± 3.5% (Les-4367 1 μM) and 83.2% ± 3.5% (Les-4367 5 μM); and Les-4367 with pertuzumab, 83.0% ± 3.4% (Les-4367 1 μM) and 87.2% ± 3.7% (Les-4367 5 μM) dead cells, respectively. In the case of the control, there were only 4.0% ± 0.3% dead cells.

### 2.3. Anti-HER2 Antibodies (Trastuzumab, Pertuzumab) Increase the Anti-Proliferative Effect of a Novel 4-Thiazolidinone Derivative (Les-4367) in AGS Gastric Cancer Cells Compared with Agents Used Alone

**Figure 2:**
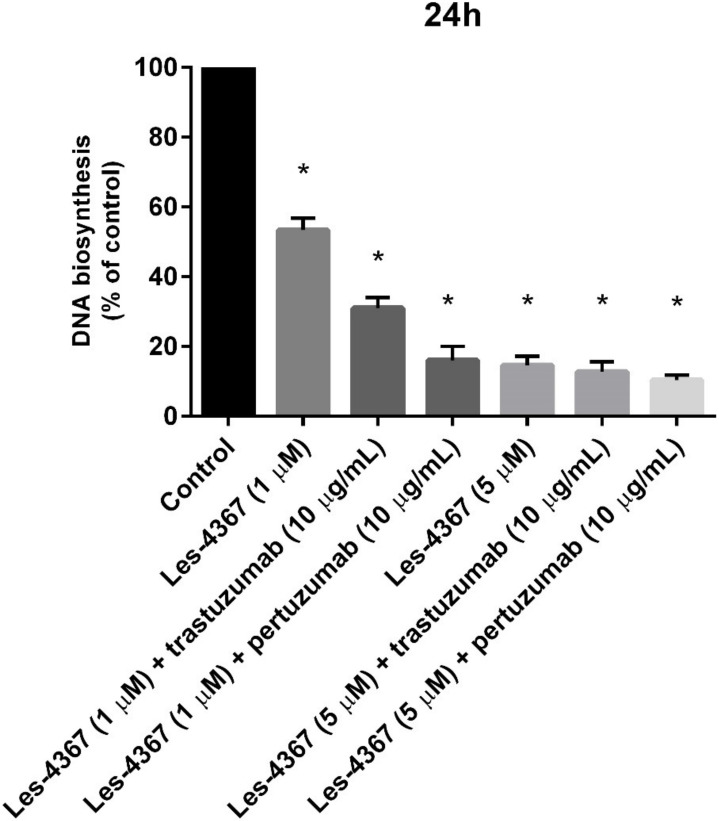
The antiproliferative effects of Les-4367 alone and combined with trastuzumab or pertuzumab (trastuzumab + Les-4367, pertuzumab + Les-4367) in cultured AGS cells after 24 h incubation, as measured by inhibition of [3H]-thymidine incorporation into DNA. Mean ± SD from three independent experiments (n = 3) done in duplicate are presented. The ANOVA and Tukey tests were used to demonstrate differences between the control cells and the cells exposed to varying concentrations of the tested compounds. * *p* ≤ 0.05 vs. control group.

The antiproliferative activity of the new 4-thiazolidinone derivative (Les-4367) was checked after 24 h of incubation, and the results are presented in Figure 2. The combination of trastuzumab or pertuzumab with the novel compound (Les-4367) strongly inhibited the proliferation of human AGS gastric cancer cells (Figure 2). After 24 h exposition of AGS cells to a lower dose of Les-4367 (1 μM) and trastuzumab (10 μg/mL), we observed that DNA biosynthesis was inhibited by 31.5%. The increase in the dose of Les-4367 to 5 μM led to a stronger inhibition of the analyzed process of 12.8%. Pertuzumab more effectively increased the susceptibility of human AGS gastric cancer cells to Les-4367. After 24 h of incubation with Les-4367 (1 μM) and pertuzumab (10 μg/mL), we demonstrated that DNA biosynthesis decreased to 16.2%. The higher dose of Les-4367 inhibited the proliferation of gastric cancer cells to 10.4%.

### 2.4. Induction of Apoptosis

**Figure 3:**
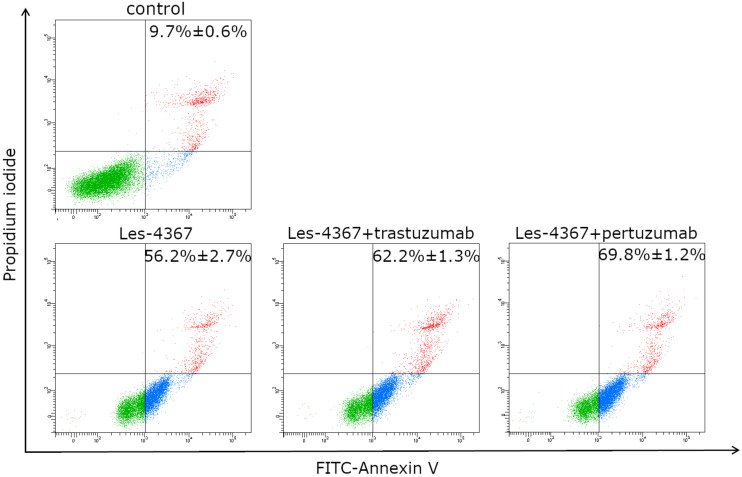
Flow cytometry analysis of AGS human gastric cancer cells after 24 h incubation with Les-4367 alone and combined with trastuzumab or pertuzumab (Les-4367 + trastuzumab, Les-4367 + pertuzumab) and subsequent staining with Annexin V and propidium iodide. The numerical value in the upper part of the dot plot = the percentage of cells with early and late apoptosis. Green part of dot plot- live cells, blue part of dot plot- cells with early apoptosis, red part of dot plot- cells with late apoptosis, gray part of dot plot- necrotic cells. The obtained results are presented as mean values ± SD obtained from three independent experiments (n = 3) done in duplicate.

For quantitative determination of the apoptotic effects of Les-4367 alone and in combination with trastuzumab and pertuzumab on human AGS gastric cancer cells, a flow cytometric assay was performed using double-staining annexin V-FITC and propidium iodide (AV/PI). The results showed that Les-4367 alone, at a concentration of 1 μM (incubation for 24 h), induced apoptosis in 56.2% ± 2.7% of AGS cells (Figure 3). In the case of combination therapy, we observed: Les-4367 with trastuzumab, 62.2% ± 1.3% (Les-4367 1 μM); and Les-4367 with pertuzumab, 69.8% ± 1.2% (Les-4367 1 μM) cells with apoptosis, respectively.

### 2.5. Trastuzumab or Pertuzumab Combined with Les-4367 Increase Caspase-8 Initiator Activity in AGS Gastric Cancer Cells Compared with the Agent Used Alone

**Figure 4:**
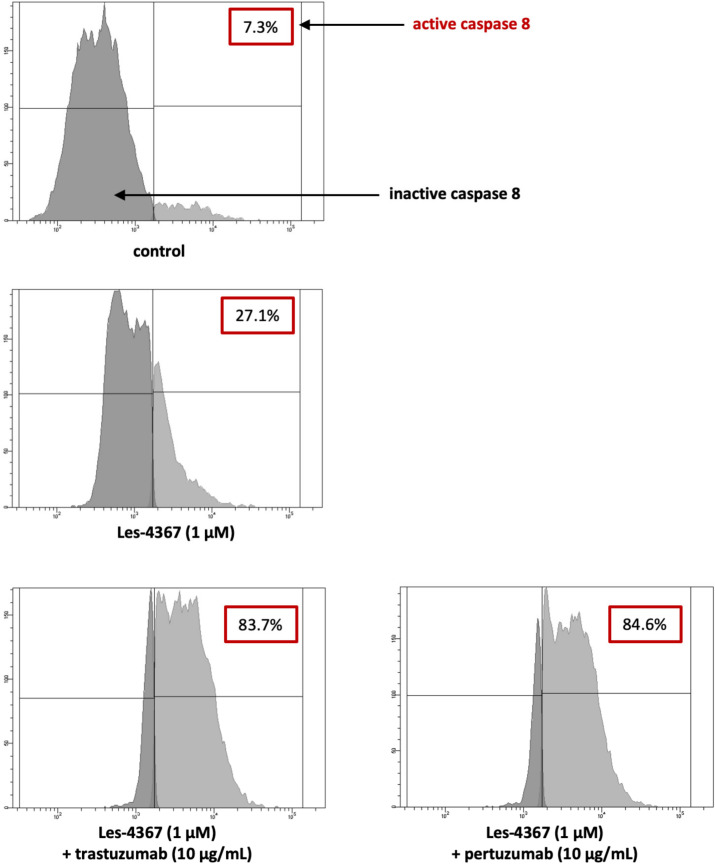
Activity of caspase-8 in AGS cells after 24 h incubation with Les-4367 (1 μM) and combined with anti-HER2 antibodies. Dark grey part of histogram- cells with inactive form of caspase-8, light grey part of histogram- cells with active form of caspase-8. The ANOVA and Tukey tests were used to demonstrate differences between the control cells and the cells exposed to varying concentrations of the tested compounds.

We examined the effect of the novel 4-thiazolidinone derivative Les-4367 (1 μM) alone and in combination with trastuzumab or pertuzumab in AGS gastric cancer cells after 24 h of incubation. As presented in Figure 4, after exposing cancer cells to the tested compound, we observed 27.1% of the cell population with active caspase-8. Combining the drug with trastuzumab or pertuzumab affects the high activity of the initiator caspase. We proved that there were 83.7% of cells with active caspase-8 after exposition to Les-4367 and trastuzumab and 84.6% with active caspase-8 after incubation with Les-4367 and pertuzumab.

### 2.6. Trastuzumab or Pertuzumab Combined with Les-4367 Increase Caspase-9 Initiator Activity in AGS Gastric Cancer Cells Compared with the Agent Alone

**Figure 5:**
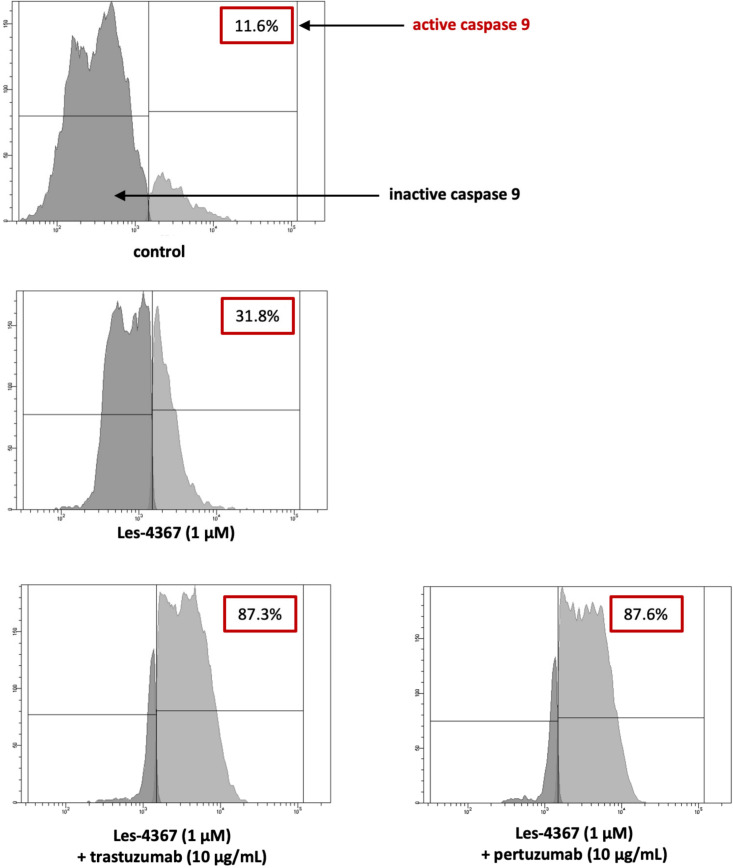
Activity of caspase-9 in AGS cells after 24 h incubation with Les-4367 (1 μM) and combined with anti-HER2 antibodies. Dark grey part of histogram- cells with inactive form of caspase-9, light grey part of histogram- cells with active form of caspase-9. The ANOVA and Tukey tests were used to demonstrate differences between the control cells and the cells exposed to varying concentrations of the tested compounds.

We also confirmed that monotherapy as well as combination therapy based on Les-4367 and anti-HER2 monoclonal antibodies led to the activation of caspase-9 in AGS gastric cancer cells (Figure 5). The exposition of cells to trastuzumab or pertuzumab with Les-4367 (1 μM) resulted in the most significant activation of caspase-9 (87.3% and 87.6%) compared with monotherapy.

### 2.7. Autophagy

**Figure 6:**
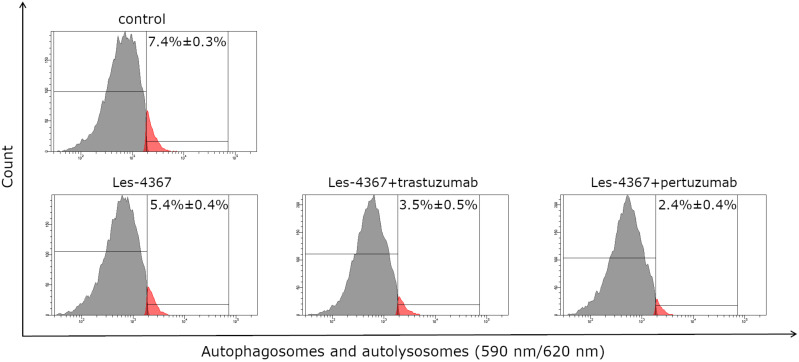
Flow cytometry analysis of autophagy induction in AGS human gastric cancer cells after 24 h incubation with Les-4367 alone and combined with trastuzumab or pertuzumab (Les-4367 + trastuzumab, Les-4367 + pertuzumab). The numerical value in the upper part of the dot plot = the percentage of cells with autophagy. Grey part of histogram- non-autophagic cells, red part of histogram- autophagic cells. The obtained results are presented as mean values ± SD obtained from three independent experiments (n = 3) done in duplicate.

To quantify the effect of Les-4367 alone and in combination with trastuzumab and pertuzumab on the autophagy process in human AGS gastric cancer cells, a flow cytometric assay was performed using ICT’s Autophagy Assay, Red. The results showed that Les-4367 alone, at a concentration of 1 μM (incubation for 24 h), induced the autophagy process in 5.4% ± 0.3% of AGS cells (Figure 6). In the case of combination therapy, we observed: Les-4367 with trastuzumab, 3.5% ± 0.5% (Les-4367 1 μM); and Les-4367 with pertuzumab, 2.4% ± 0.4% (Les-4367 1 μM) cells with autophagy, respectively. The percentage values were lower than in the control sample (7.4% ± 0.3%).

### 2.8. Trastuzumab or Pertuzumab Combined with Les-4367 Decrease Beclin-1, LC3A, and LC3B Concentrations in AGS Gastric Cancer Cells

**Figure 7:**
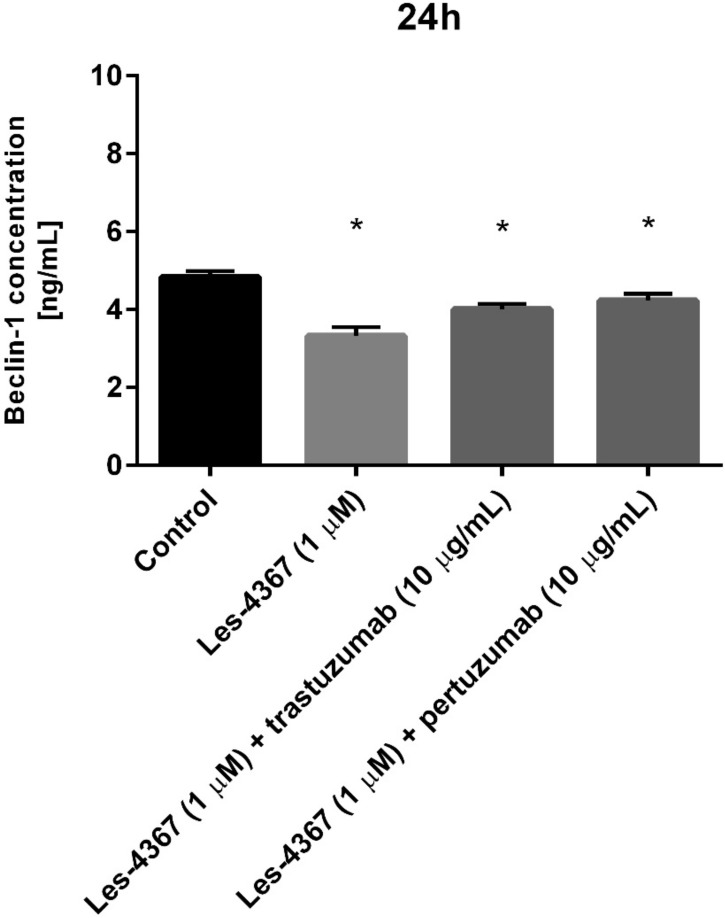
Concentrations of Beclin-1 in AGS cells after 24 h incubation with Les-4367 (1 μM) and combined with anti-HER2 antibodies. The ANOVA and Tukey tests were used to demonstrate differences between the control cells and the cells exposed to varying concentrations of the tested compounds. * *p* ≤ 0.05 vs. control group.

Analysis of the obtained results revealed that Les-4367 caused a reduction in Beclin-1 concentrations in the tested human gastric cancer cell line. As shown in Figure 7, the Beclin-1 concentration was 3.333 ng/mL after the incubation of AGS cells with 1 µM of Les-4367 compared with the control (4.833 ng/mL of Beclin-1). In the case of a combination of drugs, the concentration of the analyzed protein was 4.01 ng/mL after incubation with 1 µM of Les-4367 and trastuzumab. Similarly, we detected 4.235 ng/mL after exposition to the tested combination (1 µM Les-4367 + 10 µg/mL pertuzumab) compared with the control (4.833 ng/mL). The obtained results showed that Les-4367 alone, as well as in combination with trastuzumab or pertuzumab, decreased Beclin-1 levels in the gastric cancer cell line.

**Figure 8:**
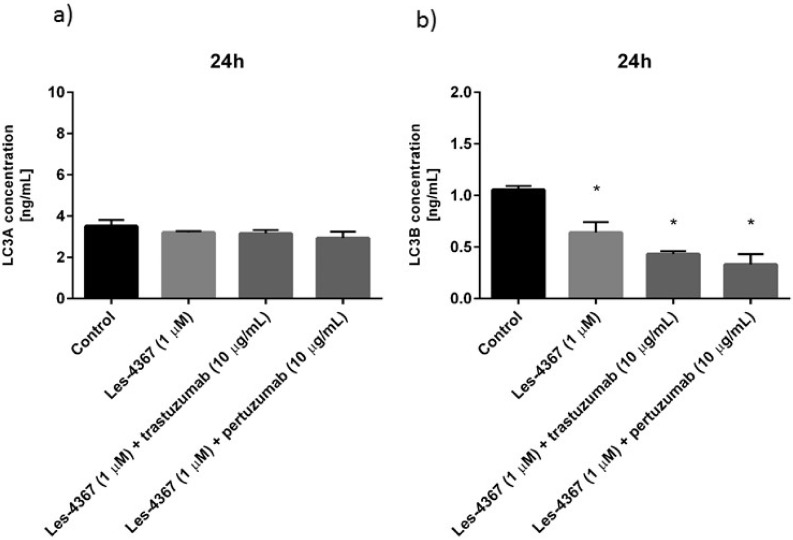
Concentrations of LC3A (**a**) and LC3B (**b**) in AGS cells after 24 h incubation with Les-4367 (1 μM) and combined with anti-HER2 antibodies. The ANOVA and Tukey tests were used to demonstrate differences between the control cells and the cells exposed to varying concentrations of the tested compounds. * *p* ≤ 0.05 vs. control group.

To investigate the effect of Les-4367 alone and combined with anti-HER2 monoclonal antibodies on the autophagy process, microtubule-associated protein 1A/1B light chain 3A (LC3A) and microtubule-associated protein 1A/1B light chain 3B (LC3B) concentrations were checked. The results are presented in Figure 8. The human AGS gastric cancer cell line was exposed to Les-4367 (1 µM) for 24 h. As demonstrated in Figure 8a, a decrease in LC3A concentration was observed after the incubation of cancer cells with the newly synthesized compound. The concentration of LC3A in untreated control AGS cells was 3.514 ng/mL. After 24 h of incubation, the novel compound Les-4367 decreased LC3A to 3.210 ng/mL (1 µM).

Similar effects were observed in the case of LC3B concentration. As demonstrated in Figure 8b, the concentration of LC3B in untreated control AGS cells was 1.054 ng/mL. The novel compound significantly reduced LC3B concentration to 0.641 ng/mL in comparison with the control (1 µM). Both combinations were more effective in decreasing LC3B concentrations than Les-4367 alone. We proved that Les-4367 (1 µM) together with trastuzumab decreased LC3B levels to 0.43 ng/mL. The combination of Les-4367 with pertuzumab was also effective in reducing LC3B concentration in cell lysates. After 24 h of incubation of human gastric cancer cells with the tested combination, the concentration of LC3B was 0.33 ng/mL (1 µM of Les-4367 + pertuzumab).

### 2.9. Trastuzumab or Pertuzumab Combined with Les-4367 Decrease ATG5 and LC3B Expression in AGS Gastric Cancer Cells

**Figure 9:**
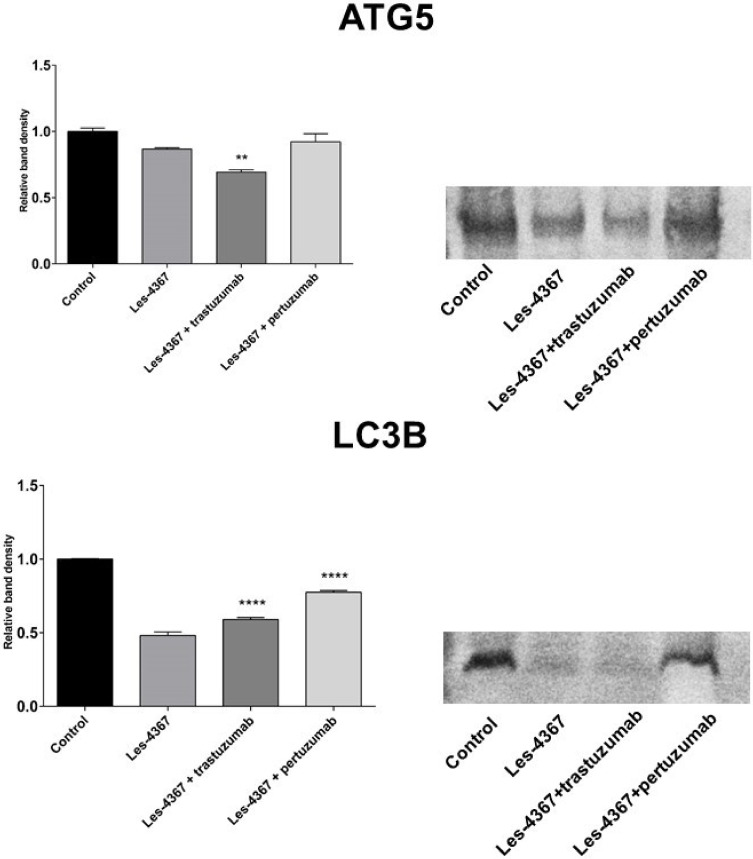
Western blot analyses of ATG5 and LC3B expression in AGS cells after 24 h incubation with Les-4367 (1 µM), Les-4367+ trastuzumab (1 µM + 10 µg/mL), and Les-4367+ pertuzumab (1 µM + 10 µg/mL). The results are presented as mean optical density ± SD from three measurements. Statistical significance was calculated using one-way ANOVA with Bonferroni multiple comparison test. Differences were considered statistically significant at ** (*p* ≤ 0.005) and **** (*p* ≤ 0.0001).

To assess how Les-4367 alone and in combination with anti-HER2 antibodies (trastuzumab and pertuzumab) affect the expression of proteins involved in autophagy processes (ATG5 and LC3B), the Western blot technique was used. Western blot analysis was performed to confirm the results obtained by the ELISA technique. Monotherapy and the combination of Les-4367 and anti-HER2 led to a reduction in ATG5 and LC3B expression in AGS cell lysates compared with the untreated control cells (Figure 9).

### 2.10. Trastuzumab or Pertuzumab Combined with Les-4367 Decrease MMP-2 and ICAM-1 Concentrations in AGS Gastric Cancer Cells

**Figure 10:**
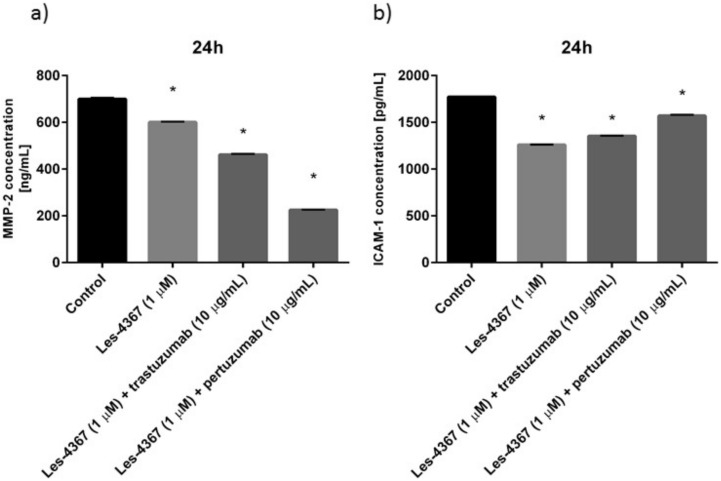
Concentrations of MMP-2 (**a**) and ICAM-1 (**b**) in AGS cells after 24 h incubation with Les-4367 (1 μM) and combined with anti-HER2 antibodies. The ANOVA and Tukey tests were used to demonstrate differences between the control cells and the cells exposed to varying concentrations of the tested compounds. * *p* ≤ 0.05 vs. control group.

MMP-2 concentration was analyzed after 24 h of incubation with the tested agents (Figure 10a). A significant reduction in MMP-2 concentration was observed after treatment with Les-4367 as well as after combination, compared with the untreated control (700 ng/mL). The most inhibitory effect was detected after 24 h of incubation with pertuzumab and Les-4367—the concentration was 225.59 ng/mL. 

Intercellular adhesion molecule-1 (ICAM-1) is a cell adhesion molecule and plays the main role in numerous immune responses [21]. Jung et al. proved that the increased expression of intercellular adhesion molecule-1 (ICAM-1) in gastric cancer could be related to the aggressive nature of the tumor and has a poor prognostic effect on gastric cancer [22]. We demonstrated that its level decreased after 24 h exposition to Les-4367 with trastuzumab, and the concentration reached 358.5 pg/mL compared with the untreated control, where ICAM-1 concentration was 481 pg/mL (Figure 10b).

### 2.11. Trastuzumab or Pertuzumab Combined with Les-4367 Exert Anti-Inflammatory Potential in AGS Gastric Cancer Cells

**Figure 11:**
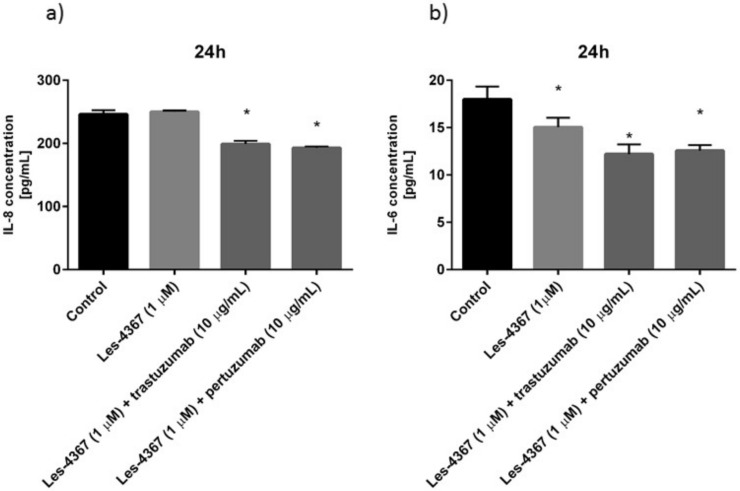
Concentration of pro-inflammatory IL-8 (**a**) and IL-6 (**b**) in AGS cells after 24 h incubation with Les-4367 (1 μM) and combined with anti-HER2 antibodies. The ANOVA and Tukey tests were used to demonstrate differences between the control cells and the cells exposed to varying concentrations of the tested compounds. * *p* ≤ 0.05 vs. control group.

**Figure 12:**
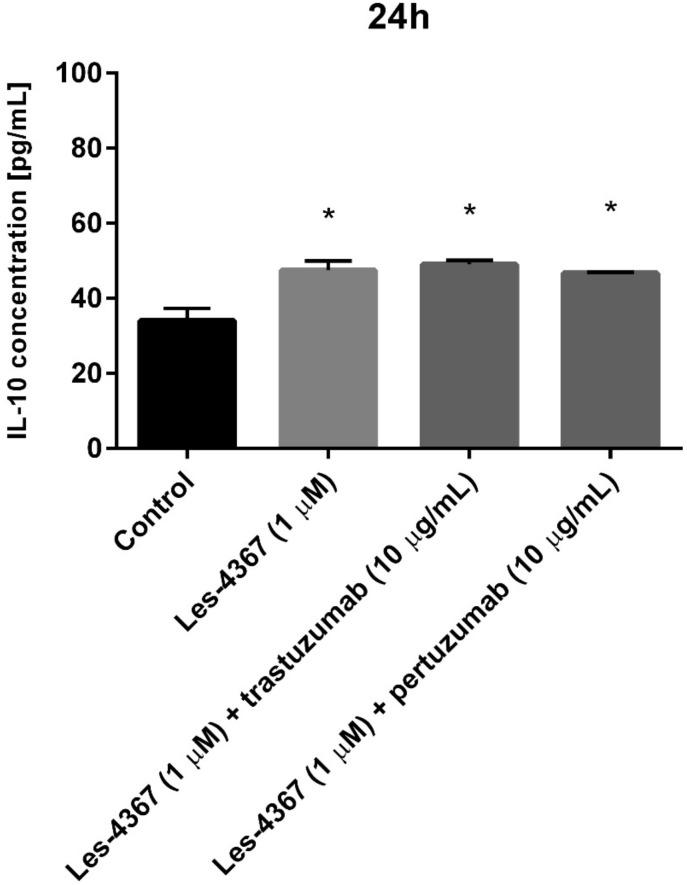
Concentrations of anti-inflammatory IL-10 in AGS cells after 24 h incubation with Les-4367 (1 μM) and combined with anti-HER2 antibodies. The ANOVA and Tukey tests were used to demonstrate differences between the control cells and the cells exposed to varying concentrations of the tested compounds. * *p* ≤ 0.05 vs. control group.

The effect of the tested Les-4367 and its combination with pertuzumab or trastuzumab on the process of inflammation was analyzed. The results are presented in Figure 11 and Figure 12.

The examined combinations with anti-HER2 antibodies led to a decrease in the analyzed cytokine concentrations. The most significant reduction in interleukin-8 (IL-8) concentration was observed after incubation with Les-4367 (1 µM) and pertuzumab and reached 193 pg/mL, compared with the control (246.33 pg/mL) (Figure 11a). To confirm the anti-inflammatory activity of the tested combinations, we checked interleukin-6 (IL-6) concentrations. We proved that the most significant reduction in IL-6 concentration was observed after 24 h incubation with Les-4367 (1 µM) and trastuzumab (10 µg/mL). We detected 12.212 pg/mL of IL-6 compared with the control sample, where the concentration of IL-6 was 17.99 pg/mL (Figure 11b). 

Figure 12 illustrates that the enhancement of interleukin-10 (IL-10) concentration was observed for monotherapy as well as both tested combinations of drugs.

In comparison with the control cells (34.03 pg/mL), the greatest increase in IL-10 concentration was detected for Les-4367 combined with trastuzumab (49.076 pg/mL). Twenty-four-hour incubation with 1 µM of Les-4367 resulted in the detection of IL-10 concentration at 47.49 pg/mL. For Les-4367 and pertuzumab, the IL-10 concentration was 46.728 pg/mL.

## 3. Discussion

Despite progress in gastric cancer treatment, the median overall survival for metastatic gastric cancer remains less than one year. The development of multi-target drugs may improve survival and reduce side effects for patients. The standard first-line therapy for HER2-positive gastric cancer is trastuzumab with chemotherapy [23]. Pertuzumab with chemotherapy was evaluated in HER2-positive metastatic gastric cancer or gastroesophageal junction cancer, but the results from the phase III JACOB trial were unsatisfactory [24,25]. Pembrolizumab is the first approved immunotherapy agent, and its mechanism of action is based on preventing the interaction of programmed cell death protein-1 (PD-1) with program cell death ligand-1 (PD-L1) and program cell death ligand- 2 (PDL-2) and restoring anti-tumor immunity [23]. Many drugs as well as combination strategies are still under investigation (anti-VEGF, small-molecule tyrosine kinase inhibitors (TKIs), anti-mTOR, anti-HGF/MET, PARP inhibitors, and immunotherapeutic agents) [23]. The development of novel drugs with promising activity against gastric cancer cells is a challenge for scientists and provides the possibility for novel, beneficial treatment strategies. 

Recently, we examined the activity of a combination of etoposide with trastuzumab or pertuzumab in AGS gastric cancer cells. The interaction of etoposide with pertuzumab or trastuzumab induced programmed cell death via extrinsic and intrinsic apoptotic pathways in AGS gastric cancer cells but did not affect autophagy, where a decrease in Beclin-1, LC3A, and LC3B was observed compared with the untreated control [26]. The preliminary results were the basis for deeper analysis. In this study, we investigated the molecular mechanisms of action of a new 4-thizolidinone derivative (Les-4367) in combination with trastuzumab or pertuzumab in the same in vitro model. 

In this study, the cytotoxic and antiproliferative potential of monotherapy as well as combination therapy was confirmed. The combination of pertuzumab with Les-4367 was the most effective in decreasing viability and DNA biosynthesis in AGS gastric cancer cells. 

Cancer cell apoptosis may be disrupted in several ways, causing neoplastic cells to proliferate in an uncontrolled way. Caspases are a group of enzymes that play a central role in the process of programmed cell death and are an important subject of research [27]. Caspase-8 is engaged in the extrinsic apoptotic pathway, and caspase-9 is a part of the intrinsic apoptotic pathway. In our research, we proved that combinations of a novel derivative with pertuzumab or trastuzumab exerted high pro-apoptotic activity. We detected significantly increased caspase-8 and caspase-9 activity compared with monotherapy. Such increased activity of initiator caspases is associated with the induction of extrinsic and intrinsic apoptotic pathways in AGS gastric cancer cells.

A potential pathway for the relationship between apoptosis and autophagy is autophagy suppression and apoptosis induction. There are a number of autophagy inhibitors undergoing clinical trials. Chloroquine is undergoing preclinical studies for metastatic prostate cancer and non-small cell lung cancer (NSCLC) treatment. Hydroxychloroquine, verteporfin, and clarithromycin are under clinical investigation [28]. FV-429 exerts a dual ability to induce apoptosis and autophagy blockage. It was examined in gastric cancers or T-cell malignancies [29,30]. In our research, we focused on relevant proteins involved in the autophagy process: Beclin-1, LC3A, LC3B, and ATG5. Trastuzumab or pertuzumab in combination with Les-4367 decreased Beclin-1, LC3A, and LC3B concentrations in AGS gastric cancer cells. The results were confirmed by Western blot analysis as well as flow cytometry. The expression of ATG5 as well as LC3B was lower than in the control sample. The molecular mechanism of action of the tested combinations was not associated with autophagy induction.

There are other known compounds that induce apoptosis, inhibit autophagy, and exert promising anticancer potential in in vitro models. Recently, the anticancer effects of new 7-methyl-5-phenyl-pyrazolo [4,3-e]tetrazolo[4,5-b[1,2,4]triazine sulfonamide derivatives were demonstrated. MM124 and MM137 decreased the concentrations of LC3A, LC3B, and Beclin-1 in the tested DLD-1 and HT-29 colon cancer cell lines, but strongly induced programmed cell death [31].

Some of the earliest observations contribute to our knowledge about the relationship between cytokine overexpression and gastric carcinogenesis. Giraud et al. demonstrated that signal transducer and activator of transcription 3 (STAT3) is responsible for gastric cancer initiation and progression as a result of its activation by cytokines (IL-6, IL-11) [32]. Additionally, in *Helicobacter pylori*-positive gastric mucosa, IL-1β, IL-6, and IL-8 mRNA expression levels correlated significantly with activity and chronic inflammation scores [33]. The development of drugs that can directly inhibit inflammation is one purpose of these studies. We checked the effect of the tested combinations of drugs on IL-6 and IL-8 concentrations in AGS gastric cancer cells. The most significant reduction in IL-6 and IL-8 concentrations was observed after the exposition of AGS gastric cancer cells to a combination of trastuzumab and Les-4367, compared with monotherapy and a combination of pertuzumab with Les-4367.

MMP-2 is considered to be a prognostic marker in patients with gastric cancer [34]. To our knowledge, MMP-2 expression was more frequent in aggressive gastric cancers and related to high cyclooxygenase-2 (COX-2) expression [35]. We proved that Les-4367 combined with pertuzumab was the most effective in reducing MMP-2 concentrations.

Researchers have reported that elevated intercellular adhesion molecule-1 (ICAM-1) expression in cancer cells and increased release of serum ICAM-1 from cancer cells are connected to cancer progression [36,37,38]. Kang et al. emphasized that ICAM-1 expression was positively correlated with various stages of gastric or hepatic cancers and could be a promising target for disease prevention and treatment [39]. In our study, a significant reduction in ICAM-1 concentration in AGS gastric cancer cells was observed after 24 h of incubation with trastuzumab and novel Les-4367.

## 4. Materials and Methods

### 4.1. Compounds

Trastuzumab and pertuzumab were the products of Selleckchem (Planegg, Germany). The purity of trastuzumab was 99.7%, and the purity of pertuzumab was 99.17%.

The new 4-thiazolidinone derivative Les-4367 was synthesized following the protocol reported in [20].

All reagents and solvents were purchased from commercial suppliers and were used directly without further purification. Melting points were measured in open capillary tubes on a BÜCHI B-545 melting point apparatus (BÜCHI Labortechnik AG, Flawil, Switzerland) and were uncorrected. The elemental analyses (C, H, and N) were performed using the Perkin-Elmer 2400 CHN analyzer (PerkinElmer, Waltham, MA, USA) and were within ±0.4% of the theoretical values. The 500 MHz ^1^H and 126 MHz ^13^C NMR spectra were recorded on a Varian Unity Plus 500 (500 MHz) spectrometer (Varian Inc., Paulo Alto, CA, USA). All spectra were recorded at room temperature, except where indicated otherwise, and were referenced internally to solvent reference frequencies. Chemical shifts (δ) are quoted in ppm and coupling constants (J) are reported in Hz. LC-MS spectra were obtained on a Finnigan MAT INCOS-50 (Thermo Finnigan LLC, San Jose, CA, USA). The reaction mixture was monitored by thin-layer chromatography (TLC) using commercial glass-backed TLC plates (Merck Kieselgel 60 F_254,_ Darmstadt, Germany). Commercially available solvents and reagents were used without further purification.

(Z)-3-(5-((5-(2-hydroxyphenyl)-3-(4-methoxyphenyl)-4,5-dihydro-1H-pyrazol-1-yl)methylene)-4-oxo-2-thioxothiazolidin-3-yl)phenyl acetate (Les-4367)

Yield 73 %, mp > 272–274 °C. ^1^H NMR (500 MHz, DMSO-d6, δ): 10.36 (s, 1H, OH), 7.83 (d, J = 8.6, 2H, arom), 7.53 (t, J = 8.1 Hz, 1H, arom), 7.48 (s, 1H, CH), 7.44 (s, 1H, arom), 7.39 (d, J = 8.4, 1H, arom), 7.23 (d, J = 8.4, 1H, arom), 7.19–7.10 (m, 5H, arom), 6.84 (d, J = 8.5 Hz, 1H, arom), 5.69 (dd, J = 11.6, 6.4 Hz, 1H, pyrazoline), 3.86 (dd, J = 18.0, 11.3 Hz, 1H, pyrazoline), 3.85 (s, 3H, CH_3_), 3.51 (dd, J = 18.0, 6.4 Hz, 1H, pyrazoline), 2.26 (s, 3H, CH_3_).

^13^C NMR (126 MHz, DMSO-d6, δ): δ 193.8 (C=S, C2), 168.9 (C=O, Ac), 165.9 (C=O, C4), 161.8, 160.0, 154.9, 150.7, 137.0, 133.3, 132.7, 131.7, 129.7, 129.2, 127.6, 126.4, 122.5, 118.3, 114.7, 110.2 (C5), 92.8 (CH, pyrazoline), 61.5 (CH_3_, OMe), 55.6 (CH_2_, pyrazoline), 20.9 (CH_3_, Ac). LCMS (ESI): m/z 546.0 (100.00%, [M + H]^+^). Anal. Calcd. for C_28_H_23_N_3_O_5_S_2_: C, 61.64%; H, 4.25%; N, 7.70%. Found: C, 61.80%; H, 4.40%; N, 7.90%.

### 4.2. Cell Culture

AGS-CRL-1739 human gastric cancer cells were obtained from the American Type Culture Collection (ATCC). AGS cells were cultured in Dulbecco’s Modified Eagle’s Medium (DMEM, Corning, Kennebunk, ME, USA), which was supplemented with 10% fetal bovine serum (FBS, Eurx, Gdansk, Poland) and a 1% cocktail of penicillin and streptomycin (Corning, Kennebunk, ME, USA). Cells were seeded in Costar flasks (Merck, Darmstadt, Germany) and grown in 5% CO2 at 37 °C to reach about 90–95% subconfluency. Then, human gastric cancer cells were treated with 0.05% trypsin and 0.02% ethylenediaminetetraacetic acid (EDTA, Corning, Kennebunk, ME, USA) in calcium-free phosphate-buffered saline, counted in a hemocytometer, and seeded in 6-well plates (Nunc) at 5 × 10^5^ cells/well in 2 mL of growth medium (DMEM). Cells that reached about 80% confluency were used for further analysis.

### 4.3. Viability

BD Horizon Fixable Viability Stain (BD Biosciences, San Diego, CA, USA) was used for the viability assay of AGS gastric cancer cells treated with Les-4367 alone (1.0 µM and 5 µM) and in combination with trastuzumab or pertuzumab (Les-4367 (1.0 µM and 5 µM) + trastuzumab (10 µg/mL) or Les-4367 (1.0 µM and 5 µM) + pertuzumab (10 µg/mL)). After 24 h of incubation with the tested compounds, AGS (adherence-deficient) gastric cancer cells were suspended in 1 mL of PBS (Corning, Kennebunk, ME, USA) solution. They were then subjected to staining with BD Horizon Fixable Viability Stain reagent for 15 min at room temperature. After this time, the cells were washed twice with BD Pharmigen Stain Buffer and resuspended in stain buffer. Thus prepared samples were immediately analyzed by a FACSCanto II flow cytometer (10,000 measured events) with FACSDiva software (both from BD Biosciences Systems, San Jose, CA, USA). The equipment was calibrated with BD Cytometer Setup and Tracking Beads (BD Biosciences, San Diego, CA, USA).

### 4.4. [^3^H]thymidine Incorporation Assay

The effect of the studied compounds—Les-4367 as well as the combination of Les-4367 (1 μM, 5 μM) with trastuzumab or pertuzumab (10 μg/mL)—on cell proliferation was tested. AGS-CRL-1739 cells were seeded in 6-well plates and cultured as described above. Cells were treated with different concentrations of the tested compounds and 0.5 μCi of [^3^H]thymidine for 24 h at 37 °C. The cells were harvested by trypsinization and washed several times in cold phosphate-buffered saline—PBS—(10 min/1.500 g) until the dpm in the washes were similar to the reagent control. Radioactivity was determined by liquid scintillation counting. [^3^H]thymidine uptake was expressed as dpm/well.

### 4.5. Apoptosis

AGS human gastric cancer cells were incubated with Les-4367 alone (1 µM) and in combination with trastuzumab or pertuzumab (Les-4367 (1 µM) + trastuzumab (10 µg/mL); Les-4367 (1 µM) + pertuzumab (10 µg/mL)) for 24 h and stained with Annexin V-FITC/PI. The analysis was performed using flow cytometry Apoptosis Detection Kit II (BD Biosciences, San Diego, CA, USA) as previously described [40]. Gastric cancer cells (AGS) were incubated for 24 h (37 °C, 5% CO_2_, 90–95% humidity) with the tested compounds. First, in the cells treated with the tested compounds as well as the controls, the medium was removed and the cells were washed twice with cold PBS. Subsequently, cells were resuspended in the binding buffer included in the kit at a concentration of 1 × 10^6^ cells/mL. From each sample, 100 µL of cell suspension was taken and transferred to test tubes, into which 5 µL each of FITC Annexin V and propidium iodide (PI) were then added. The contents of the test tubes were gently vortexed and incubated for 15 min at room temperature, protected from light. After the required time, the contents of the test tubes were made up to 500 µL with binding buffer and immediately analyzed in a flow cytometer (10,000 events measured). After the flow cytometer readout, the results were analyzed using FACSDiva software (BD Biosciences Systems, San Jose, CA, USA). The equipment was calibrated with BD Cytometer Setup and Tracking Beads (BD Biosciences, San Diego, CA, USA).

### 4.6. Caspase-8 Enzymatic Activity Assay

The FAM-FLICA Caspase-8 Kit (ImmunoChemistry Technologies, Davis, CA, USA) was used to determine caspase-8 activity after treating AGS-CRL-1739 cells with Les-4367 (1 μM) as well as a combination of Les-4367 (1 μM) with trastuzumab or pertuzumab (10 μg/mL) for 24 h. After incubation, the cells were harvested and washed with cold PBS. Then, 5 μL of diluted FLICA reagent and 2 μL of Hoechst 33342 were added to 93 μL of the cell suspension and mixed by pipetting. The gastric cancer cells were incubated for 60 min at 37 °C. After that time, the cells were washed twice in 400 μL apoptosis wash buffer and centrifuged at 300× *g*. After the last wash, the cells were resuspended in 100 μL of apoptosis wash buffer and supplemented with 10 μg/mL PI. Analysis was performed using the BD FACSCanto II flow cytometer, and the obtained results of the study were analyzed with FACSDiva software (both from BD Biosciences Systems, San Jose, CA, USA).

### 4.7. Caspase-9 Enzymatic Activity Assay

The FAM-FLICA Caspase-9 Kit (ImmunoChemistry Technologies, Davis, CA, USA) was used to analyze caspase-9 activity, measured according to the manufacturer’s instructions. The AGS-CRL-1739 cells were exposed to Les-4367 (1 μM) with trastuzumab or pertuzumab (10 μg/mL) for 24 h. After that, the cells were harvested and washed with cold PBS. FLICA reagent (5 μL) and Hoechst 33342 (2 μL) were added to the cell suspension (93 μL) and mixed by pipetting. The cells were incubated for 60 min at 37 °C, then washed twice using a wash buffer and centrifuged at 300× *g*. After the last wash, the cells were resuspended in 100 μL of apoptosis wash buffer and supplemented with 10 μg/mL PI. A BD FACSCanto II flow cytometer was used to perform the result analysis.

### 4.8. Autophagy

The effect of Les-4367 alone (1 µM) and in combination with trastuzumab or pertuzumab (Les-4367 (1 µM) + trastuzumab (10 µg/mL), Les-4367 (1 µM) + pertuzumab (10 µg/mL)) on the induction of autophagy in human AGS gastric cancer cells was assessed by ICT’s Autophagy Assay, Red, using a flow cytometer. The test was performed as previously described [26]. Briefly, cells were washed with PBS and suspended in 490 µL of PBS. To each probe, 10 µL of diluted Autophagy Probe Red solution was added. The prepared samples were incubated at 37 °C for 60 min. Following incubation, the cells were suspended in diluted cellular assay buffer and centrifuged. Then, the supernatant was removed, and the cells were resuspended in diluted cellular assay buffer and centrifuged again. This step was repeated three times. Finally, the cell pellet was resuspended in 0.5 mL of diluted cellular assay buffer. Thus, prepared samples were immediately analyzed by a FACSCanto II flow cytometer (10,000 measured events) with FACSDiva software (both from BD Biosciences Systems, San Jose, CA, USA). The equipment was calibrated with BD Cytometer Setup and Tracking Beads (BD Biosciences, San Diego, CA, USA).

### 4.9. Concentrations of Beclin-1, IL-8 and IL-6, IL-10 and ICAM-1

High-sensitivity human Beclin-1, IL-8, and IL-6 SimpleStep ELISA (abcam, Cambridge, UK) kits were used to determine the protein concentrations in cell lysates from the AGS cell culture after 24 h of incubation with anti-HER2 monoclonal antibodies, Les-4367 (1 μM), as well as a combination of Les-4367 (1 μM) with trastuzumab or pertuzumab (10 μg/mL). Briefly, the trypsinized cells were washed three times with cold PBS and centrifuged at 1000× *g* for 5 min at 4 °C. The cells (1.5 × 10^6^) were suspended in lysis buffer for whole cell lysates. After centrifugation, the supernatants were frozen immediately at -80 °C. The standards, samples, and antibody cocktail were added to the appropriate microtiter plate wells. After 1 h of incubation at room temperature, the microplate wells were aspirated and washed three times, and then TMB development solution was added to each well. The enzyme-substrate reaction was terminated by adding stop solution, and the color change was measured spectrophotometrically at a wavelength of 450 nm ± 2 nm. The antigen concentrations in the samples were determined by comparing the O.D. of the samples to the standard curve.

### 4.10. ATG5 and LC3B Expression

Cell lysate samples containing 30 μg of protein each were subjected to SDS-PAGE. The electrophoresis was run at 100 V for 1.5 h. Protein transfer to nitrocellulose membranes was done in the electrophoresis unit (1 h at 20 mA). After the transfer, nitrocellulose was washed with 5% non-fat dairy milk in TBS-T (TRIS-buffered saline with Tween 20 (20 mM TRIS-HCl buffer, pH 7.6, with 150 mM NaCl and 0.05% Tween 20)) for 1 h. Subsequently, overnight incubation of membranes with monoclonal antibodies against ATG5 and LC3B in TBS-T took place. Then, secondary alkaline phosphatase-conjugated antibodies against rabbit immunoglobulin (1:1000) diluted in TBS-T were added to each nitrocellulose membrane, followed by 1 h of incubation with gentle shaking. After the incubation, the nitrocellulose membranes were washed with TBS-T four times and exposed to Sigmafast BCIP/NBT (Merck, Darmstadt, Germany) in the darkness. Images of the nitrocellulose membranes were subsequently captured and analyzed.

### 4.11. Concentrations of LC3A, LC3B, and MMP-2

High-sensitivity assay kits (EIAab, Wuhan, China) were used to determine the concentrations of proteins in cell lysates and media from the AGS cell culture after 24 h of incubation with the tested compounds. Briefly, the trypsinized cells were washed three times with cold PBS and centrifuged at 1000× *g* for 5 min at 4 °C. The cells (1.5 × 10^6^) were suspended in lysis buffer for whole cell lysates. After centrifugation, the supernatants were frozen immediately at −80 °C. The microtiter plate provided in this kit was pre-coated with an antibody specific to the analyzed antigen. The standards and samples were added to the appropriate microtiter plate wells. After 2 h of incubation at 37 °C, the plate was incubated with biotin-conjugated antibody for 1 h at 37 °C. Then, the microplate wells were aspirated, washed three times, and incubated with avidin conjugated to horseradish peroxidase (HRP). Next, a TMB substrate solution was added to each well. Those wells that contained the target antigen exhibited a change in color. The enzyme-substrate reaction was terminated by adding a sulfuric acid solution, and the color change was measured spectrophotometrically at a wavelength of 450 nm ± 2 nm. The antigen concentrations in the samples were determined by comparing the O.D. of the samples to the standard curve.

### 4.12. Statistical Analysis

The study results are presented as the mean ± standard deviation (SD) from three independent experiments. Statistical analysis was performed using GraphPad Prism Version 6.0 (San Diego, CA, USA). The ANOVA and Tukey tests were used to demonstrate differences between the control cells and the cells exposed to varying concentrations of the tested compounds. A statistically significant difference was defined as *p* < 0.05.

## 5. Conclusions

Our research fits perfectly into the trend of targeted therapies in cancer. The molecular mechanism of action of Les-4367 is associated with the induction of extrinsic and intrinsic apoptotic pathways, decreased MMP-2 and ICAM-1 concentrations—which are involved in the invasion of gastric cancer—as well as reduced levels of the pro-inflammatory cytokine IL-6. We demonstrated for the first time that the combination of a novel 4-thiazolidinone derivative (Les-4367) with anti-HER2 antibodies (trastuzumab and pertuzumab) is a promising anticancer strategy in AGS gastric cancer cells. Based on the obtained results, it can be concluded that the molecular mechanism of action of the analyzed combinations is related to the induction of both apoptotic pathways, which was confirmed by an increase in caspase-8 and caspase-9 activity. Such combinations decreased the most significant autophagic markers, such as Beclin-1, LC3A, LC3B, and ATG5. A beneficial anti-inflammatory response was also observed. We proved that anti-HER2 antibodies with Les-4367 led to decreased IL-6 and IL-8 concentrations as well as increased IL-10 concentration. The future treatment landscape for both combinations of drugs in gastric cancer is promising.

## Figures and Tables

**Table 1:** Viability of AGS, SK-BR-3, HCC1954, MDA-MB-231, HT-29, and DLD-1 cancer cells after 24 h of Les-4367 exposure estimated by MTT assay.

Cancer Cell Lines	IC_50_
AGS	1.5 µM
SK-BR-3	9.05 µM
HCC1954	>10 µM
MDA-MB-231	3.33 µM
HT-29	8.73 µM
DLD-1	7.0 µM

## Data Availability

Not applicable.

## Supplementary Materials

The supporting information can be downloaded at: https://www.mdpi.com/article/10.3390/ijms24076791/s1.

## Abbreviations

AGS-CRL-1739	human gastric cancer cell line
Akt	protein kinase B
ANOVA	analysis of variance
ATCC	American Type Culture Collection
ATG5	autophagy related 5
AV/PI	annexin V/propidium iodide
BCIP/NBT	5-chromo-4-chloro-3-indolyl-phosphate/nitro blue tetrazolium
BMI	body mass index
COX-2	cyclooxygenase-2
DLD-1	colorectal adenocarcinoma cell line
DMEM	Dulbecco’s Modified Eagle’s Medium
DMSO	dimethyl sulfoxide
DNA	deoxyribonucleic acid
EDTA	ethylenediaminetetraacetic acid
ELISA	enzyme-linked immunosorbent assay
ERBB2	Erb-B2 receptor tyrosine kinase 2
FAM-FLICA	fluorochrome-labeled inhibitors of caspases
FBS	fetal bovine serum
FDA	Food and Drug Administration
FITC	fluorescein isothiocyanate
FV-429	flavonoid
GC	gastric cancer
HCC 1954	epithelial breast cancer cell
HER-2	human epidermal growth factor receptor 2
HGF/MET	hepatocyte growth factor/proto- oncogenic receptor tyrosine kinase
HRP	horseradish peroxidase
HT-29	human adenocarcinoma colorectal cell line
ICAM-1	intercellular adhesion molecule 1
ICT	intramolecular charge transfer
IL-1β	interleukin 1β
IL-6	interleukin 6
IL-8	interleukin 8
IL-10	interleukin 10
LC3A	microtubule-associated protein 1A/1B light chain 3A
LC3B	microtubule-associated protein 1A/1B light chain 3B
LC-MS	liquid chromatography tandem mass spectrometry
MAPK	mitogen-activated protein kinase
MCF-7	human breast cancer cell line
MDA-MB-231	triple-negative human breast cancer cell line
MMP-2	Metalloproteinase-2
^13^C NMR	carbon-13 nuclear magnetic resonance
^1^H NMR	proton nuclear magnetic resonance
NSCLC	non-small cell lung cancer
O.D.	optical density
PARP	poly (ADP-ribose) polymerase
PBS	Phosphate-buffered saline
PD-1	programmed cell death protein 1
PDL-2	program cell death ligand 2
PI	propidium iodide
PI3K	phosphatidylinositol-3 kinase
ROS	reactive oxygen species
SD	standard deviation
SDS-PAGE	sodium dodecyl-sulfate polyacrylamide gel electrophoresis
SK-BR-3	human breast cancer cell line that overexpresses the HER2 gene product
STAT3	signal transducer and activator of transcription 3
TBS-T	tris-buffered saline with tween
TKI	tyrosine kinase inhibitor
TLC	thin layer chromatography
TMB	tetramethylbenzidine
mTOR	mammalian target of rapamycin
TRIS	tris(hydroxymethyl)aminomethane
VEGF	vascular endothelial growth factor
