# Supplementary material for: Multi-Targeting Anticancer Activity of a New 4-Thiazolidinone Derivative with Anti-HER2 Antibodies in Human AGS Gastric Cancer Cells
Source: Int J Mol Sci. 2023 Apr 5;24(7):6791. doi: 10.3390/ijms24076791 (PMC10095353; doi:10.3390/ijms24076791)
Supplement: Supplementary file 1 [file ijms-24-06791-s001.zip › ijms-2317480-supplementary.pdf]

Supplementary information

Table of Contents

Copies of ^{1}H, ^{13}C NMR spectra of compound **Les-4367** S2

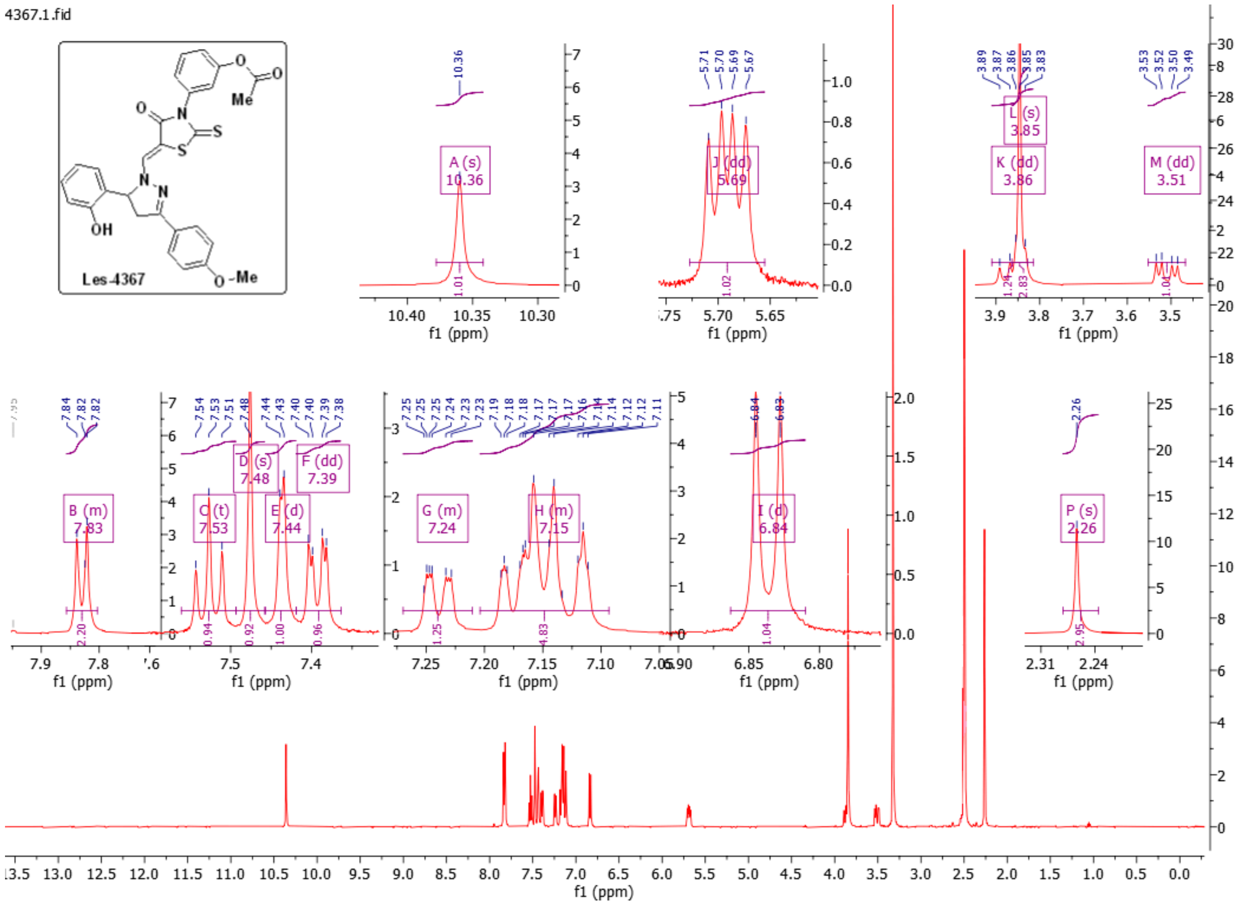

Figure S1. ^{1}H NMR spectrum of compound **Les-4367**

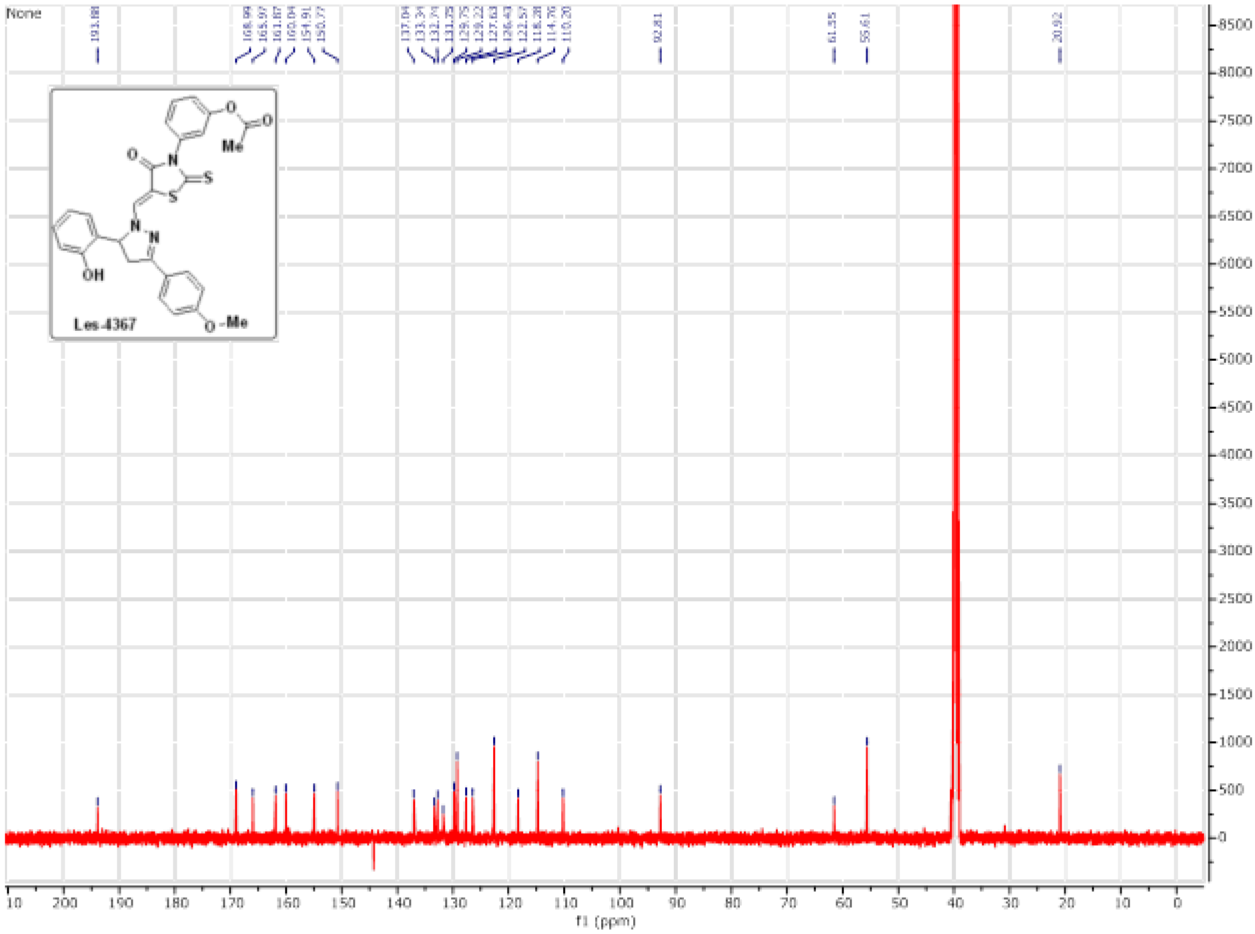

Figure S2. ^{13}C NMR spectrum of compound **Les-4367**
